# FELD better not thinking of metastases only when liver lesions appear after bleomycin-based treatment for non-seminoma testis from metastases

**DOI:** 10.1186/1471-2407-13-491

**Published:** 2013-10-22

**Authors:** Filip YFL De Vos, Sasja F Mulder, Joost PH Drenth, Iris D Nagtegaal, Jurgen J Fütterer, Winette TA van der Graaf

**Affiliations:** 1 Department of Medical Oncology, Radboud University Nijmegen Medical Centre, P.O. Box 9101, 6500 HB Nijmegen, Netherlands; 2 Department of Gastroenterology and Hepatology, Radboud University, Nijmegen Medical Centre, P.O. Box 9101, 6500 HB Nijmegen, Netherlands; 3 Department of Pathology, Radboud University Nijmegen Medical Centre, P.O. Box 9101, 6500 HB Nijmegen, Netherlands; 4 Department of Radiology, Radboud University Nijmegen Medical Centre, P.O. Box 9101, 6500 HB Nijmegen, Netherlands

**Keywords:** Transient, Eosinophilia, Liver lesions, Non-seminoma testis

## Abstract

**Background:**

Bleomycin has become an integral part of chemotherapy in patients with germ-cell tumors. One of the most feared side effects is bleomycin-induced pneumonitis. In patients with mild or moderate BIP, radiological signs disappear almost completely within nine months after discontinuation of bleomycin treatment.

**Case presentation:**

We present a patient with a history of non seminoma of the testis and bleomycin-induced pneumonitis. During follow-up, regression of the hypothesis of eosinophilic migration to the liver after regression of bleomycin-induced pneumonitis is highly suspicious based on transient eosinophilia and focal eosinophilic liver disease.

**Conclusion:**

As follow up may consist of CT scanning in germ-line tumor patients, transient eosinophilic liver lesions reported during regressive bleomycin-induced pneumonitis should not be presumed automatically as metastatic tumor relapse and require further sequential imaging and pathological examination.

## Background

Bleomycin is a glycopeptide antibiotic produced by the bacterium *Streptomyces verticillus*. Bleomycin acts as an oncolytic agent by inducing DNA strand breakage and subsequent has become an integral part of chemotherapy in patients with germ-cell tumors [1,2]. Bleomycin-induced toxicity usually targets organs with low hydrolase concentrations i.e. lungs and skin [3]. One of the most feared side effects is bleomycin-induced pneumonitis (BIP) [4]. BIP is a potential life-threatening interstitial pulmonary fibrosis. Depending on the diagnosis criteria used, up to 46% of patients treated with bleomycin develop BIP [4]. Treatment of BIP consists of discontinuation of bleomycin. In severe BIP cases, steroids are indicated, while a case-report mentions imatinib mesylate as a salvage therapy in steroid-resistant BIP [4,5]. In patients with mild or moderate BIP, radiological signs disappear almost completely within nine months after discontinuation of bleomycin treatment [6]. In this case-report, transient eosinophilia and focal eosinophilic liver lesions occurred simultaneously with regression of BIP lesions, fuelling the hypothesis of eosinophilic migration. It implicates sequential computer tomography (CT) scanning and robust pathologic evidence for diagnosing relapse of testicular cancer in such cases.

## Case presentation

**Figure 1:**
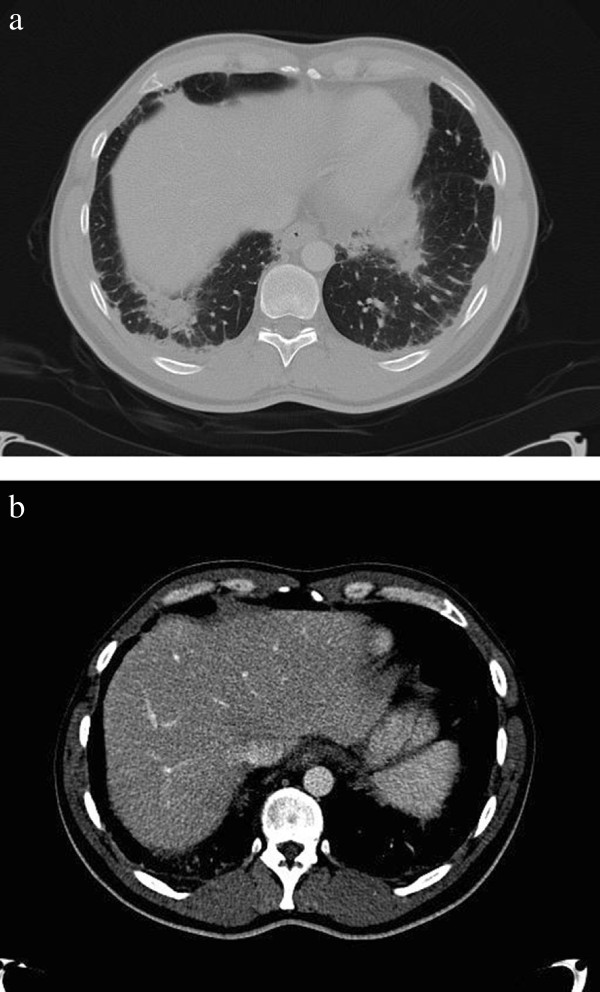
**Radiologic findings at the end of chemotherapy. (a)** Fibrosis in both lungs with signs of bronchiolitis obliterans and focally organizing pneumonia; **(b)** No signs of liver lesions.

**Figure 2:**
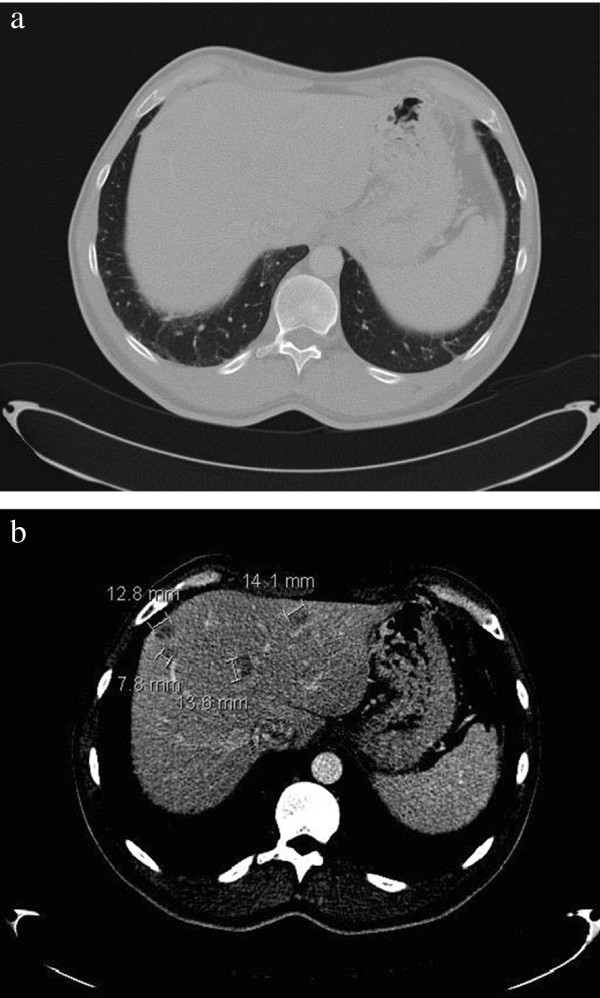
**Radiologic findings one year after end of chemotherapy. (a)** Pulmonary infiltrations resolving; **(b)** Four new hypo-dense lesions in the liver.

**Figure 3:**
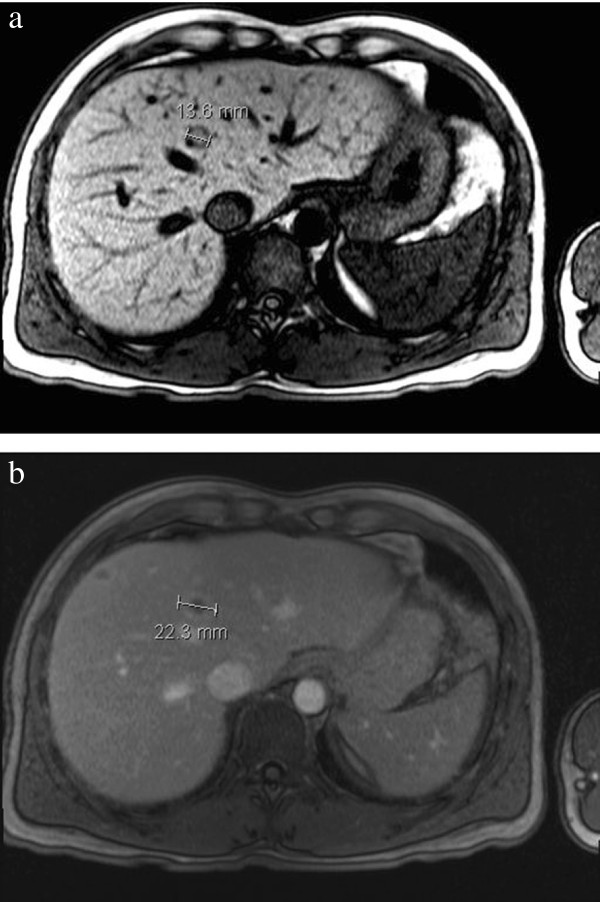
**Characterization by MRI. (a)** Lesion with hypointense rim and isointense centre; **(b)** Lesion with centrally low signal intensity and rim enhancement.

**Figure 4:**
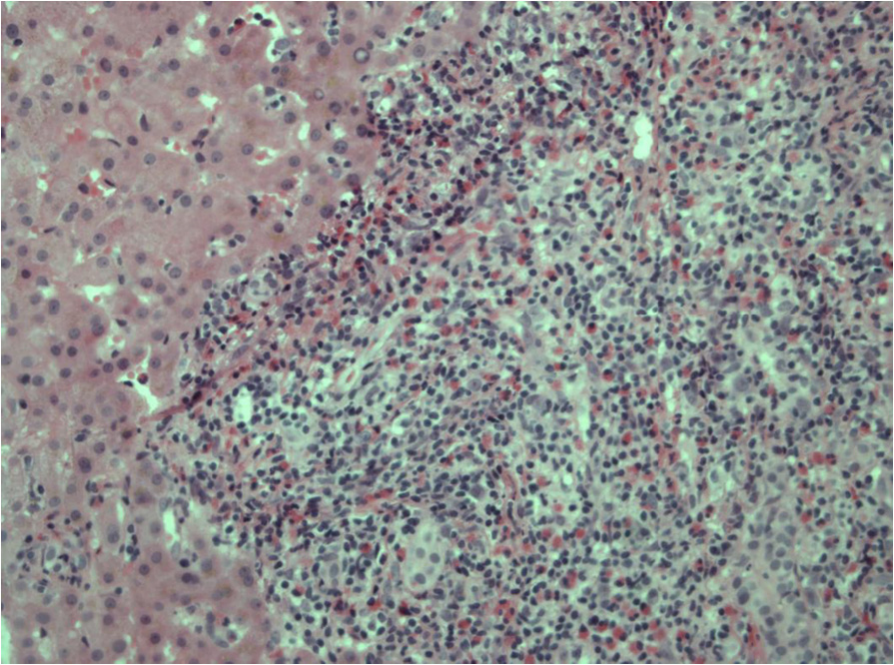
Pathologic findings of one of the liver lesions revealing portal inflammation, infiltration of eosinophils and lymphocytes with focal necrosis.

**Figure 5:**
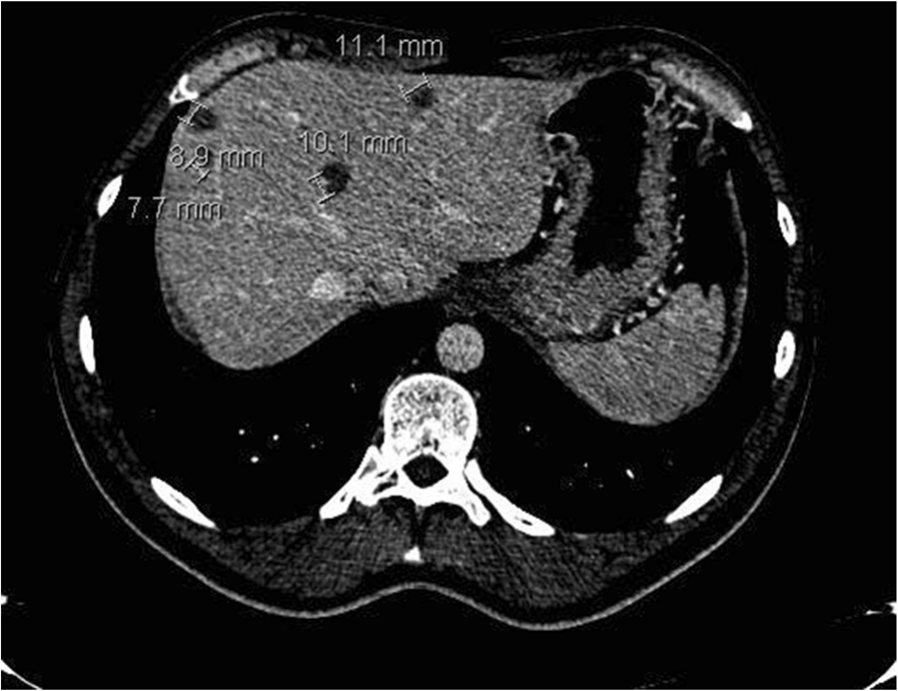
Abdominal CT-scan fifteen months after end of chemotherapy with further regression of pulmonary fibrosis, and regression of liver lesions.

A 41-year-old man was diagnosed with stage IIA good risk non seminoma of the left-sided testis and treated with hemiorchidectomy and adjuvant three cycles of bleomycin, etoposide and cisplatin. He received a total dose of 270 mg bleomycin during treatment. After the last course of chemotherapy, a chest and abdominal CT-scan (CT 1) revealed complete remission of the metastatic lesions. However, we unexpectedly discovered fibrosis in both lungs with signs of bronchiolitis obliterans and focally organizing pneumonia, probably induced by bleomycin (Figure 1a), while liver lesions were absent (Figure 1b). He had no pulmonary complaints. No broncho-alveolar lavage was performed. Our patient was closely monitored according to national guidelines [7]. A year after end of chemotherapy, with pulmonary infiltrations resolving (Figure 2a) routine CT scan (CT 4) showed four new hypo-dense lesions in the liver with a maximal diameter of 20 mm (Figure 2b. At that moment, the patient reported no complaints. Tumor markers, human chorionic gonadotropin, alpha fetoprotein levels and lactate dehydrogenase, were normal. Laboratory findings reported 7.3×109/l leucocytes with 11% eosinophils (absolute eosinophil count 0.8 × 109/l, normal value < 0.5) normal liver enzymes, bilirubin level and liver function tests (prothrombin time, albumin and glucose). The patient had no history of travel related diseases, dietary habits and other risk factors for eosinophilia. Sarcoidosis was ruled out by a normal serum angiotensin-converting enzyme. Hepatitis serology and bacteriological cultures were all negative. Additional, magnetic resonance imaging (MRI) was performed for further characterization (Figure 3a and 3b). On the non-contrast T1-weighted axial MRI image (Figure 3a) a lesion with a hypointense rim and isointense centre was seen. On the contrast enhanced T1-weighted fat-suppressed axial MR image (hepatocyte phase, Figure 3b) a lesion with centrally low signal intensity and rim enhancement suggestive for small abscess was seen. A needle biopsy of one of the liver lesions showed no signs of tumor, normal architecture of central veins and portal fields and portal inflammation with infiltration of eosinophils and lymphocytes with focal necrosis (Figure 4). Extensive discussions in our tumor panel and with our hepatology experts led to the diagnosis of bleomycin induced focal hepatitis with eosinophilic infiltration based on exclusion of other possible diagnoses, time-relationship with BIP regression and pathologic findings. A wait-and-see policy was adopted with CT scanning (CT 6) three months later (Figure 5).

**Figure 6:**
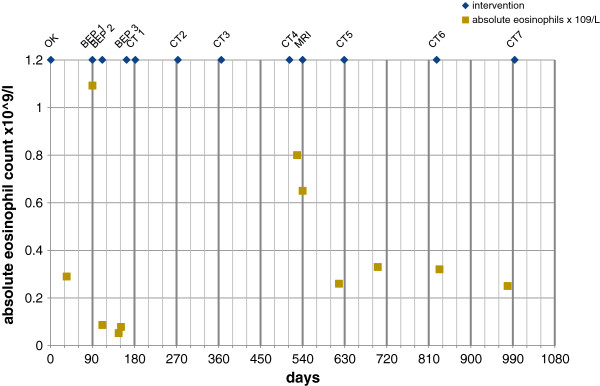
**Excel-file graph of absolute eosinophil count over time.** Absolute eosinophil count, with a normal value of < 0,5 × 109/l OK= operation orchidectomy BEP = chemotherapy course with Bleomycine, Etoposide and Cisplatin CT1 = CT scan with bilateral pulmonary infiltrations, no liver abnormalities (Figure 1) CT2 = CT scan with decreased pulmonary fibrosis, no liver abnormalities CT3 = CT scan with decreased pulmonary fibrosis, no liver abnormalities CT4 = CT scan with remains of pulmonary fibrosis and 4 liver lesions (Figure 2) MRI = MRI liver, 4 liver lesions (Figure 3a and 3b) CT5 = CT scan with remains of pulmonary fibrosis and 4 decreased liver lesions CT6 = CT scan with remains of pulmonary fibrosis and 3 liver lesions stable, 1 decreased (Figure 5) CT7 = CT scan with remains of pulmonary fibrosis and 4 liver lesions stable with central calcifications.

This CT revealed further regression of pulmonary fibrosis, and regression of liver lesions in segment 2 (n = 1) and in segment 8 (n = 3). At 10 months one lesion was further decreased 3 lesions were stable. Interestingly, subsequent blood measurements showed a normalization of the percentage eosinophils in the three months between the first occurrence of liver lesions on CT and second CT with diminishment of these liver lesions and eventually calcification. This leads to the hypothesis that both findings are related to each other (Figure 6).

## Discussion

We have seen the concomittant regression of BIP and onset of focal eosinophilic liver disease (FELD) with eosinophilia. By exclusion diagnosis and thorough pathologic examination, this relationship in time led to the hypothesis of eosinophilic migration. Our understanding of the pathogenesis of BIP is mainly based on data derived from animal studies. Endothelial damage of the lung vasculature by bleomycin-induced free radicals is associated with an acquired loss of bleomycin hydrolase activity and followed by an influx of inflammatory cells [8]. There is a significant correlation between eosinophilia and bleomycin-induced pulmonary fibrosis [9,10]. Apart from T-lymphocytes, eosinophils are key players in the production of tumor growth factor-β, platelet-derived growth factor receptor-α and tumor necrosis factor-α, leading to proliferation and accumulation of fibroblasts. On their turn, fibroblasts produce chemotactic cytokines recruiting eosinophils [11,12]. The trigger for the self-limiting nature of BIP remains elusive due to complex interaction between several T-lymphocyte related chemokines [13,14]. We hypothesize that pulmonary eosinophils and fibroblasts migrate to other organs such as the liver, leading to transient eosinophila and focal eosinophilic accumulation. FELD is a well-known disease entity that is associated with a variety of pathological conditions including parasitic infestations, allergy, internal malignancies, drug hypersensitivity, and hypereosinophilic syndrome [15,16]. FELD can be differentiated from hepatic metastases using CT or MRI scanning. A significant smaller lesion size on unenhanced T1-weighted compared to hepatocyte phase imaging (delayed phased) and an ill-defined margin and isointensity on T1 weighted images can distinguish FELD from liver hepatic metastases [17-19]. These characteristics were also observed in our patient. The biopsy-proven eosinophilic infiltrations had irregular, fuzzy margins, while arterial hyperintensity was lacking. In our patient, liver biopsy revealed typical FELD characteristics. Nevertheless, after extensive biochemical, serological and bacteriological testing, no specific cause, related to a transient appearance of eosinophilia and eosinophil infiltration in the liver, was determined. Yet, a clear time-relationship was observed between eosinophilia and the onset of pulmonary fibrosis and liver lesions and normalization of eosinophil count and regression of pulmonary fibrosis and liver lesions (Figure 6). This lead to the hypothesis of interrelated cause and effect. Focal liver lesions in patients with BIP do not necessarily imply the relapse of germcell tumors as is demonstrated in our case. It is always a challenge to differentiate between metastatic or nonmetastatic liver lesions (Table 1). It is likely that corticosteroids given for BIP, for example in case of severe pulmonary symptoms, would ameliorate the intense inflammatory reaction [4,20]. However, as corticosteroids have not direct effect on the initial inflammatory reaction leading to BIP, our patient would not have benefited and the liver lesions would probably still have emerged.

**Table 1:** Differential diagnosis between metastatic liver lesions and FELD

	**Metastatic liver lesion**	**FELD**
Incidence/100,000	8 – 20	3 – 4
Solitary	5 – 10%	90%
Pathogenesis	Hematogenous or lymphatic spread of cancer	parasitic infestations, allergy, internal malignancies, drug hypersensitivity, and hypereosinophilic syndrome
Imaging	US, CT	Two-phase dynamic CT, MRI
Alpha fetoprotein; human chorionic gonadotropin	Elevated (in case of non-seminoma testis)	Normal
Calcification	Possible	None
Characteristic gross features	Hemorrhage, necrosis with rim enhancement on CT, spherical shape	indistinct margins, absence of rim enhancement, nonspherical shape
Characteristic microscopic features	Replacement of hepatocytes, by malignant cells no portal structures	focal eosinophilic accumulation
Diagnosis	FNAB or core biopsy	FNAB or core biopsy
Treatment	Resection, RFA or chemotherapy	Depending underlying disease

*US*: ultrasound; *CT*: computerized tomography; *MRI*: magnetic resonance imaging; *FNAB*: fine needle aspiration biopsy for cytology; *RFA*: radiation frequency ablation.

## Conclusion

In this patient the hypothesis of eosinophilic migration to the liver after regression of BIP is suggestive given the transient eosinophilia and presence of FELD. As follow up may consist of CT scanning in germ-line tumor patients, transient eosinophilic liver lesions reported during regressive BIP should not be presumed automatically as metastatic tumor relapse and require further sequential imaging and pathological examination.

## Consent

Patient has given his consent for publication of case-report.

## Abbreviations

BIP: Bleomycin-induced pneumonitis; CT: Computer tomography; FELD: Focal eosinophilic liver disease; MRI: Magnetic resonance imaging.

## Competing interests

No funding sources need to be credited. No conflicts of interest should bementioned.

## Authors’ contributions

IN provided the figures of pathological findings. JF provided the figures of radiological findings. All authors read and approved the final manuscript.

## Pre-publication history

The pre-publication history for this paper can be accessed here:

http://www.biomedcentral.com/1471-2407/13/491/prepub
